# Improved spatial–temporal graph convolutional networks for upper limb rehabilitation assessment based on precise posture measurement

**DOI:** 10.3389/fnins.2023.1219556

**Published:** 2023-07-11

**Authors:** Jing Bai, Zhixian Wang, Xuanming Lu, Xiulan Wen

**Affiliations:** ^1^Industrial Technology Research Institute of Intelligent Equipment, Nanjing Institute of Technology, Nanjing, China; ^2^Jiangsu Provincial Engineering Laboratory of Intelligent Manufacturing Equipment, Nanjing, China; ^3^‘School of Automation, Nanjing Institute of Technology, Nanjing, China

**Keywords:** rehabilitation assessment, upper limb, posture measurement, graph convolutional networks, motion range

## Abstract

After regular rehabilitation training, paralysis sequelae can be significantly reduced in patients with limb movement disorders caused by stroke. Rehabilitation assessment is the basis for the formulation of rehabilitation training programs and the objective standard for evaluating the effectiveness of training. However, the quantitative rehabilitation assessment is still in the experimental stage and has not been put into clinical practice. In this work, we propose improved spatial-temporal graph convolutional networks based on precise posture measurement for upper limb rehabilitation assessment. Two Azure Kinect are used to enlarge the angle range of the visual field. The rigid body model of the upper limb with multiple degrees of freedom is established. And the inverse kinematics is optimized based on the hybrid particle swarm optimization algorithm. The self-attention mechanism map is calculated to analyze the role of each upper limb joint in rehabilitation assessment, to improve the spatial-temporal graph convolution neural network model. Long short-term memory is built to explore the sequence dependence in spatial-temporal feature vectors. An exercise protocol for detecting the distal reachable workspace and proximal self-care ability of the upper limb is designed, and a virtual environment is built. The experimental results indicate that the proposed posture measurement method can reduce position jumps caused by occlusion, improve measurement accuracy and stability, and increase Signal Noise Ratio. By comparing with other models, our rehabilitation assessment model achieved the lowest mean absolute deviation, root mean square error, and mean absolute percentage error. The proposed method can effectively quantitatively evaluate the upper limb motor function of stroke patients.

## 1. Introduction

Stroke is the second leading cause of death in the world, and its incidence rate is on the rise in recent years (Paul and Candelario-Jalil, 2021). The disability rate of this disease is high, and more than 50% of survivors will leave varying degrees of disability, which seriously affecting the daily life of patients, causing great pain to themselves, and adding a heavy economic burden to families and society. The World Stroke Organization (WSO) estimates that the global cost of stroke is over $721 billion (Feigin et al., 2022). Therefore, there is a great demand for rehabilitation training and assessment in patients with motor dysfunction.

Rehabilitation assessment is not only the basis of making a rehabilitation treatment plan but also the objective standard to observe its treatment effect. It plays an important role in rehabilitation treatment, evaluation of treatment effect, and prediction of functional recovery (Liao et al., 2020). At present, the commonly used assessment method is carried out by experienced rehabilitation physicians using the evaluation scale. The popular clinical evaluation tools are the Brunnstrom evaluation method, Fugl-Meyer Assessment (FMA), Barthel index, and so on. However, these methods are subjective assessment methods of rehabilitation physicians, with inconsistent judgment standards and inability to distinguish between compensation and true recovery (Li et al., 2022; Rahman et al., 2023). The main defect of the subjective scale is that it has a ‘ceiling effect’ on patients with mild injury. In addition, completing assessment tests is time consuming, complex, and labor intensive.

Scholars have carried out related research on rehabilitation assessment to solve the problems above. It is proposed to use an inertial measurement unit, accelerometer, VICON, infrared camera, and so on to capture human posture data (Fang et al., 2019; Hussain et al., 2019; Ai et al., 2021). The manual features are extracted from human posture data to represent human motion (Cai et al., 2019; Hamaguchi et al., 2020). Mahalanobis distance, and dynamic time warping (DTW) algorithm is used to quantify the correctness of rehabilitation exercise, support vector machine, logistic regression, and neural network are also used to grade the rehabilitation assessment (Houmanfar et al., 2016; Fang et al., 2019; Li et al., 2021). These methods rely on the results of sub-problems such as preprocessing and feature extraction, but the optimal solution of the sub-problem is not necessarily the global optimal solution and lacks end-to-end learning intuition.

Because wearable measuring equipment is very cumbersome to use, the acceptance of patients is not high, markers may be moved due to soft tissue effects, and motion capture systems such as VICON are too expensive. As an unmarked tool, Kinect is increasingly being applied to human posture tracking (Zelai and Begonya, 2016; Bawa et al., 2021). Kinect-based joint data contains a variety of information, including spatial information between joint nodes and their adjacent nodes, as well as time-domain information between frames. It has been widely used in motion recognition (Wang et al., 2020), gesture recognition (Ma et al., 2021), somatosensory interaction (Qiao et al., 2022), and also has applications in rehabilitation assessment (Agami et al., 2022) proposed a method for generating accurate skeleton data based on the offline fusion of a Kinect 3D video sensor and an electronic goniometer. This method is difficult to measure the patient’s joint angles with the electronic goniometer (Lee et al., 2018) used Kinect v2 and force sensing resistor sensors based on Fugl-Meyer assessment for evaluating upper extremity motor function (Bai and Song, 2019) conducted a preliminary rehabilitation assessment using the first-generation Kinect to measure the joint data of stroke patients, ignoring the drawbacks of a single camera.

However, there is an issue of inaccurate joint position recognition using a single Kinect. This type of erroneous recognition is prone to occur in situations of self-occlusion, when the subject is not facing the camera, or when moving at high speeds (Han et al., 2016; Wang et al., 2016). This is because although the connections of the bones obtained during recognition are biologically consistent, the length of the limbs and the limitations of the joints are not limited, resulting in unrealistic and distorted movements. Adding additional manual measurements or wearable sensors can be time-consuming and reduce patient comfort. The accuracy of tracking data for human motion posture seriously affects the correctness of rehabilitation assessment results, therefore, the accuracy of human motion tracking should be improved. How to improve the accuracy of patient pose recognition using only visual sensors is a complex problem.

An approach to improve the accuracy of human motion tracking is to combine a rigid body model with the depth camera (Matthew et al., 2019) used this approach in the sit-to-stand movement and the upper limb motion. Due to the lack of hand modeling and occlusion, the estimation of joint position is incorrect. In the Proximal Function test, the system error is introduced, and the accuracy of the overall pose estimate is reduced (Matthew et al., 2020). Using one Kinect for rehabilitation assessment (Liu et al., 2016), the body information is particularly prone to occlusion, in some specific evaluation movements such as touching the back of the head, touching the lumbar vertebrae, and so on. The occlusion problem should be solved in order to improve the accuracy of rehabilitation assessment. So, in our work, we have added an Azure Kinect and optimized the rigid body model.

Neural networks and deep learning have been used in quantitative rehabilitation assessment research (Kipf and Welling, 2017; Williams et al., 2019). Graph convolutional neural networks have been widely used in traffic prediction based on historical traffic speeds and route maps (Guo et al., 2019). It is also possible to realize action recognition and gesture recognition based on human skeleton data (Ahmad et al., 2021). According to current research, spatial–temporal graph convolutional networks (STGCN) have been used to achieve motion recognition based on dynamic bones (Yan et al., 2018). However, the application of STGCN in upper limb rehabilitation assessment is relatively limited. This study proposes to use an improved STGCN based on precise posture measurement to assess the motor function of hemiplegic upper limbs.

In this work, we proposed an innovative method as follows: two Azure Kinects is used combined with a comprehensive rigid body model to improve the biological feasibility of the skeleton. A hybrid particle swarm optimization algorithm is used to optimize inverse kinematics. A rich movement protocol is proposed to test the movement of the patient’s upper limbs from the reachable workspace and proximal function. A modified STGCN model with LSTM is proposed to assess the upper limb motor function.

## 2. Methods

**Figure 1:**
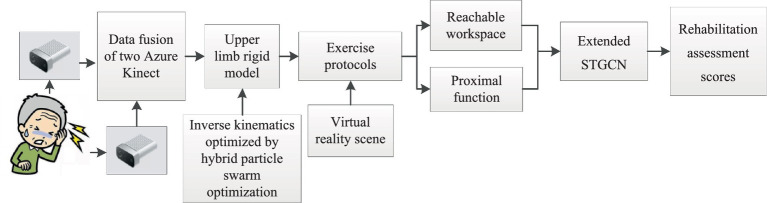
The upper limb rehabilitation assessment method based on posture measurement.

We proposed an upper limb rehabilitation assessment method based on posture measurement, as shown in Figure 1. The upper limb rigid body model is established to increase the constraints of biological behavior and improve the accuracy of human posture data collection. The motion protocol for upper limb motion assessment is proposed, and the extended STGCN is adopted to achieve continuous upper limb rehabilitation assessment.

### 2.1. Two-camera synchronization

Azure Kinect can extract the position of 32 human bone skeletons, and there is occlusion when the upper limb moves to the back of the body. Using two Azure Kinect can effectively fill the occluded area and increase the spatial coverage of the camera. Therefore, we use two Azure Kinect to collect the motion of patients’ limbs in this study. During the use of two Azure Kinect, synchronization is necessary to ensure that each frame of data captured by the two cameras is the scene at the same time. One camera is set as master and the other as subordinate. The two cameras are connected *via* a 3.5 mm synchronization port attached to the device. This study adopts a daisy chain configuration, with the master’s synchronization port connected to the output synchronization port of the slave device through a cable. Then calibrate the two devices using the black and white checkerboard calibration method to obtain the internal and external parameters of the devices, and fuse joint data from different perspectives into the same perspective.

### 2.2. Models

**Figure 2:**
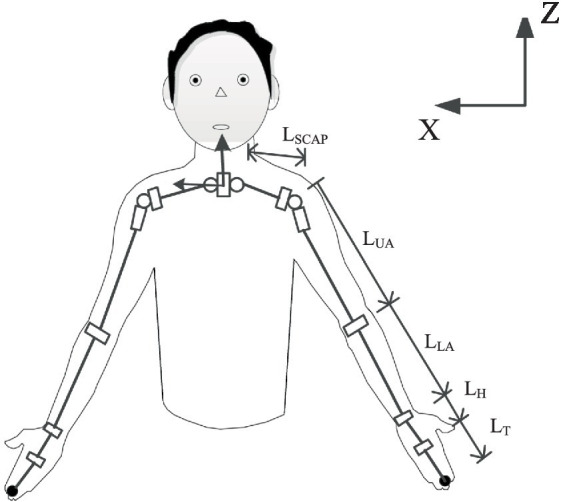
The rigid model of upper limb.

Taking the torso as the base frame, the upper limbs can be modeled as two branches of the torso. The kinematic model of the right arm is as follows. The Kinect captured joints information, the Torso can be defined by the spine-chest and spine-naval markers. Anatomically, the shoulder is a complex composed of the glenohumeral joint, sternoclavicular joint, acromioclavicular joint and, the scapulothoracic joint. The glenohumeral joint can realize flexion/extension, adduction/abduction, and adduction/abduction. The sternoclavicular joint allows retraction/protraction, elevation/depression and backward of the glenohumeral joint. The elbow allows two movements for flexion/extension and pronation/supination. To simplify the human upper limb mechanism model, this paper singles out 2-DOF at the sternoclavicular joint, 3-DOF at the shoulder, 1-DOF at the elbow, 2-DOF at the wrist, and 1-DOF at the hand. Thus, the equivalent mechanism model of human upper limbs can be established as a 9-DOF series motion model, as shown in Figure 2. $L_{SCAP}$ is the initial length of the upper limb girdle, $L_{UA}$ is the length of the upper arm, $L_{LA}$ is the length of the forearm, $L_{H}$ is the length from palm to the wrist, $L_{T}$ is the length of the hand tip. The position of the hand, wrist, elbow, shoulder, clavicle, neck, and spine chest can be obtained by Azure Kinect. The base frame is fixed at the neck and the hand position is the palm position. Both left and right hands are modeled, and the right hand is taken as an example to illustrate the modeling process.

#### 2.2.1. Rigid model

The upper limb rigid model consists of 10 segments connected by 11 joint markers. The human torso is modeled as the base of the rigid body, the neck joint of the torso is set as the origin, and the two scapulae rotate at the origin. The rigid body model is divided into two continuous chains of the left arm and the right arm. The motion of the torso (T) in the world coordinate system (W) is modeled as a system with associated homogeneous transformations:


(1) $$T_{W,To}=\left[\begin{array}{cc} R_{X}R_{Y}R_{Z} & t\\ 0 & 1 \end{array}\right]$$

Where R represents rotation, each rotation is determined by the angle θ, and t represents translation. Then model the scapula (SC), upper arm (UA), forearm (FA), hand (HA) and fingertip (TIP) as two branches of the trunk. The right arm is modeled as:


(2) $$T_{TO,RSC}=\left[\begin{array}{cc} R_{Z}R_{Y} & 0\\ 0 & 1 \end{array}\right]$$

(3) $$T_{RSC,RUA}=\left[\begin{array}{cc} R_{X}R_{Y}R_{Z} & q_{RSJC}\\ 0 & 1 \end{array}\right]$$

(4) $$T_{RUA,RFA}=\left[\begin{array}{cc} R_{X} & q_{REJC}\\ 0 & 1 \end{array}\right]$$

(5) $$T_{RFA,RHA}=\left[\begin{array}{cc} R_{X}R_{Z} & q_{RHJC}\\ 0 & 1 \end{array}\right]$$

(6) $$T_{RHA,RTIP}=\left[\begin{array}{cc} R_{X} & q_{RTJC}\\ 0 & 1 \end{array}\right]$$

The left arm is modeled in a similar way, but the direction of rotation is opposite. The forward kinematics model of the rigid body can be obtained by multiplying the coordinate changes of each segment in turn. The positions of each joint can be written as:


(7) $$\begin{array}{c} p_{tor}=T_{W,TO}\left(t,\theta \right)q_{tor}\\ p_{RSHO}=T_{W,RSC}\left(t,\theta \right)q_{RSHO}\\ \begin{array}{c} p_{RELB}=T_{W,RUA}\left(t,\theta \right)q_{RELB}\\ \begin{array}{c} p_{RWRI}=T_{W,RFA}\left(t,\theta \right)q_{RWRI}\\ \begin{array}{c} p_{RHAN}=T_{W,RHA}\left(t,\theta \right)q_{RHAN}\\ p_{RTIP}=T_{W,RTIP}\left(t,\theta \right)q_{RTIP} \end{array} \end{array} \end{array} \end{array}$$

Where *q* is the local position of each joint. The position of each joint *p* is solved according to the forward kinematics, and the mapping relationship is established. The forward kinematic map is:


(8) $$F\left(x_{i}\right)=\left[\begin{array}{l} p_{TOR},\;p_{RSHO},\;p_{RELB},\;p_{RWRI},\;p_{RHAN},\;p_{RTIP},\\ p_{LSHO},\;p_{LELB},\;p_{LWRI},\;p_{LHAN},\;p_{LTIP} \end{array}\right]$$

Scapulohumeral rhythm is present during arm abduction (Klopčar and Lenarčič, 2006) calculated the scapulohumeral rhythm of the generalized shoulder joint movement of the upper limb on four lifting planes with angles of 0°, 45°, 90° and 135° through experiments. The functional relationship between the lift angle β and the forward/backward extension angle $\theta_{fb}$ and the upward/downward angle $\theta_{ud}$ of the SC joint is as follows.


(9) $$\theta_{fb}=\{\begin{array}{cc} -0.35\beta & \beta <0{}^{\circ}\\ 0{}^{\circ} & 0{}^{\circ}\leq \beta \leq 70{}^{\circ}\\ -0.22\beta +15.4{}^{\circ} & \beta >70{}^{\circ} \end{array}$$

(10) $$\theta_{ud}=\{\begin{array}{cc} -0.3\beta & \beta <0{}^{\circ}\\ 0{}^{\circ} & 0{}^{\circ}\leq \beta \leq 30{}^{\circ}\\ 0.36\beta -10.8{}^{\circ} & \beta >30{}^{\circ} \end{array}$$

#### 2.2.2. Inverse kinematics optimized by crossbreed particle swarm optimization

The inverse kinematics of the rigid body model is a nonlinear problem. Solving the joint posture through the upper limb end posture is a one-to-many mapping relationship. The inverse kinematics is optimized based on a hybrid particle swarm optimization algorithm. The classical particle swarm optimization (PSO) algorithm belongs to a global random optimization algorithm with the advantages of few parameters required, simple algorithm structure, fast operation speed, etc. (Zhou et al., 2011). Suppose a D dimension search space has N particles, the position, and velocity of a particle in a group is,


(11) $$\begin{array}{l} X_{i}={\left[x_{i1},x_{i2},\cdots ,x_{iD}\right]}^{T}\\ V_{i}={\left[v_{i1},v_{i2},\cdots v_{iD}\right]}^{T} \end{array}$$

The evolution of particles at each iteration consists of three parts: inheritance of the previous velocity, self-memory, and information exchange of the population. Therefore, the kth iteration process can be expressed as:


(12) $$\begin{array}{c} v_{ij}\left(k+1\right)=\omega v_{ij}\left(k\right)+c_{1}r_{1}\left[p_{\text{best}}\left(k\right)-x_{ij}\left(k\right)\right]\\ \;+c_{2}r_{2}\left[g_{\text{best}}\left(k\right)-x_{ij}\left(k\right)\right] \end{array}$$

(13) $$x_{ij}\left(k+1\right)=x_{ij}\left(k\right)+v_{ij}\left(k+1\right)$$

Where ω is inertia weight coefficient, c_1_ and c_2_ are two different learning factors, r_1_ and r_2_ are two randomly generated numbers in [0,1], $p_{\text{best}}$ represents the personal best solution of the particle, $g_{\text{best}}$ represents the global best solution of the swarm.

Due to the drawbacks of premature convergence and poor local optimization ability in PSO. Crossbreed Particle Swarm Optimization (CBPSO) is used to increase the fitness of the offspring population through the natural evolution of the population, thus jumping out of the local extreme value in the search process and converging to the global optimal solution. During the iteration process, the formula for updating the position and velocity of the offspring particles is as follows:


(14) $$\{\begin{array}{c} \text{child}\left(x\right)=P_{c}\times {\text{parent}}_{1}\left(x\right)+\left(1-P_{c}\right)\times {\text{parent}}_{2}\left(x\right)\\ \text{child}\left(v\right)=\frac{{\text{parent}}_{1}\left(v\right)+{\text{parent}}_{2}\left(v\right)}{\left|{\text{parent}}_{1}\left(v\right)+{\text{parent}}_{2}\left(v\right)\right|}\left|{\text{parent}}_{1}\left(v\right)\right| \end{array}$$

where child(x) and child(v) represent the position and velocity of the child particle respectively, parent(x) and parent(v) represent position and the velocity of the parent particle, respectively. When two particles trapped in different local optimums are hybridized, they can often escape from the local optimality, and the introduction of a hybrid algorithm can enhance the global optimization ability of the population.

Our goal is to make the “distance” between the current end effector position $F\left(x_{i}\right)$ and $y_{k}$ the shortest. So inverse kinematics is transformed into an optimization problem.$y_{k}$ is the joint point collected by Azure Kinect. The fitness function is as follows:


(15) $$\text{fitness}\left(X^{*}\right)=\min{\left\|y_{k}-F\left(x_{i}\right)\right\|}^{2}$$

The specific steps of the algorithm are: First initialize the particle swarm and parameter settings, and then iterate the algorithm to calculate the fitness function value of each particle, compare the current fitness of each particle with the size of the individual extreme value, update the individual extreme value, and judge whether the hybridization condition is met. If not, return to the continuous update. Finally, select the particles corresponding to the global extremum as the optimal solution for the population.

### 2.3. Extended STGCN

**Figure 3:**
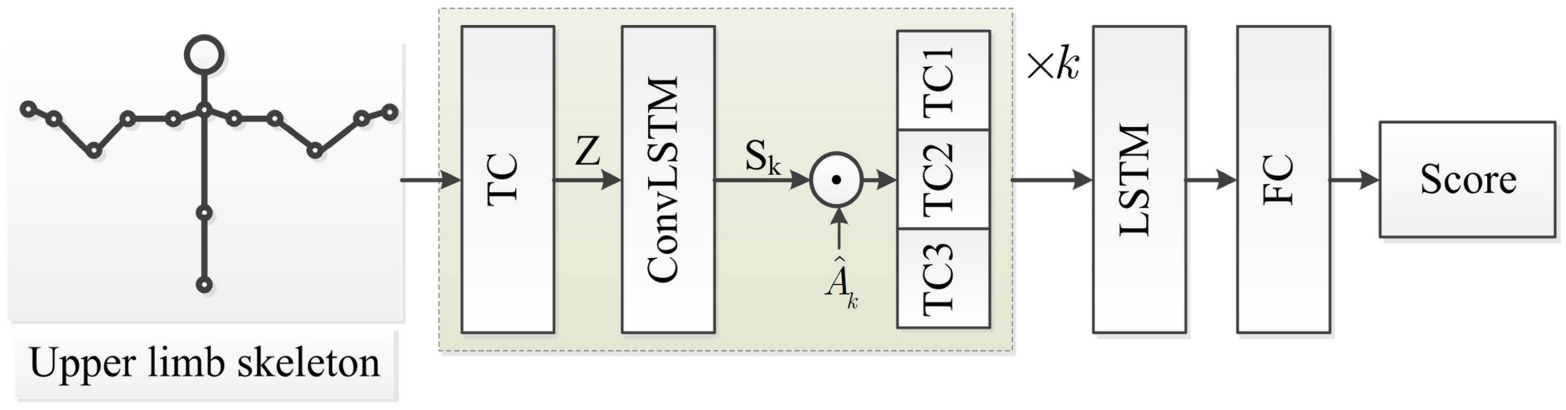
The extended STGCN for rehabilitation exercise assessment.

Human skeleton motion is a string of time series, with spatial features at each time point and time features between frames. In the process of evaluating the motor function of the upper limb, different joints play different roles. For example, in the movement of touching the nose with the right upper limb, the joints on the left side of the body participate less and show less importance, and the degree of participation of the joints on the right side is different. Self-attention mechanisms can select more critical information from a lot of information. The self-attention mechanism is adopted to extract the spatial relationship of each joint and distinguish their important degree, in order to guide patients to strengthen the rehabilitation training of important joints and obtain higher evaluation scores. The extended graph network structure is shown in Figure 3.

ConvLSTM can extract the characteristics of spatial and temporal features on time series data simultaneously (Deb et al., 2022). The STGCN is improved by the self-attention mechanism graph $S_{k}$ calculating form ConvLSTM. The skeleton sequence is initially processed by temporal convolution with kernel $\Gamma^{\mu }$.


(16) $$Z=X⊕\left(\Gamma^{u}⊗V\right)$$

Where, V is the skeleton sequence, let $Q_{k}=ZW_{k}$


(17) $$\begin{array}{c} \;i_{k}^{t}=\sigma \left(W_{i}*Q_{k}^{t}+U_{i}*h_{k}^{t-1}+b_{i}\right)\;\\ \;f_{k}^{t}=\sigma \left(W_{f}*Q_{k}^{t}+U_{f}*h_{k}^{t-1}+b_{f}\right)\\ \begin{array}{c} o_{k}^{t}=\sigma \left(W_{o}*Q_{k}^{t}+U_{o}*h_{k}^{t-1}+b_{o}\right)\\ \;g_{k}^{t}=\tanh\left(W_{c}*Q_{k}^{t}+U_{c}*h_{k}^{t-1}+b_{c}\right)\\ \begin{array}{c} c_{k}^{t}=f_{k}^{t}⊙c_{k}^{t-1}+i_{k}^{t}⊙g_{k}^{t}\\ h_{k}^{t}=o_{k}^{t}⊙\tanh\left(c_{k}^{t}\right) \end{array} \end{array} \end{array}$$

Where ∗ is convolution, σ is the sigmoid function, W is weight, b is bias, $S_{k}=h_{k}$. The GCN improved with the self-attention map is as follows,


(18) $$G_{k}=\sigma \left(\phi \left({\hat{A}}_{k}⊙S_{k}\right)ZW_{k}\right)$$

(19) $${\hat{A}}_{k}=A_{k}+I$$

Where, $A+I=\underset{k}{\sum }A_{k}$, $A\in R^{N\times N}$ is the adjacency matrix, $A_{0}=I$ and $A_{1}=A$, D is the degree matrix, $W_{k}$ is the weight matrix. ϕ is normalization, σ is an activation function.

Then three Temporal convolutional (TC) layers with different kernels $\Gamma_{1}^{l}$, $\Gamma_{2}^{l}$ and $\Gamma_{3}^{l}$ is adopted to extract time features and concatenate them. Multiple STGCN layers are stacked to obtain more complex features of different lengths, and LSTM is used to extract the time dependence of the series. Finally, continuous rehabilitation assessment scores are obtained by the full connection layer.

### 2.4. Exercise protocols base on VR

The exercise protocols were designed according to the anatomical position, clinical evaluation methods such as the Fugl-Meyer scale, Barthel index, range of motion, and some related articles. The measurement of upper limb motor function mainly includes distal reachable workspace measurement and proximal function measurement, the specific movement methods are shown in Table 1. The reachable workspace measurement was used to evaluate the motion range of the upper limb, and the proximal functional measurement was used to evaluate the subjects’ ability to self-care in daily life, such as eating, combing hair, and so on.

**Table 1:** The exercise protocols.

IDX	Protocol					
Reachable workspace (straighten the arm)	Vertical direction	Azimuth angle (°)	0	45	90	135
		Elevation angle (°)	0 ~ 180			
	Horizontal direction	Azimuth angle (°)	0 ~ 135			
		Elevation angle (°)	45	90	135	0 ~ 180
Proximal function	①Side, ②Lumber spine, ③Stomach, ④Contralateral shoulder, ⑤Ipsilateral shoulder, ⑥Nose, ⑦Ear, ⑧Head top					

**Figure 4:**
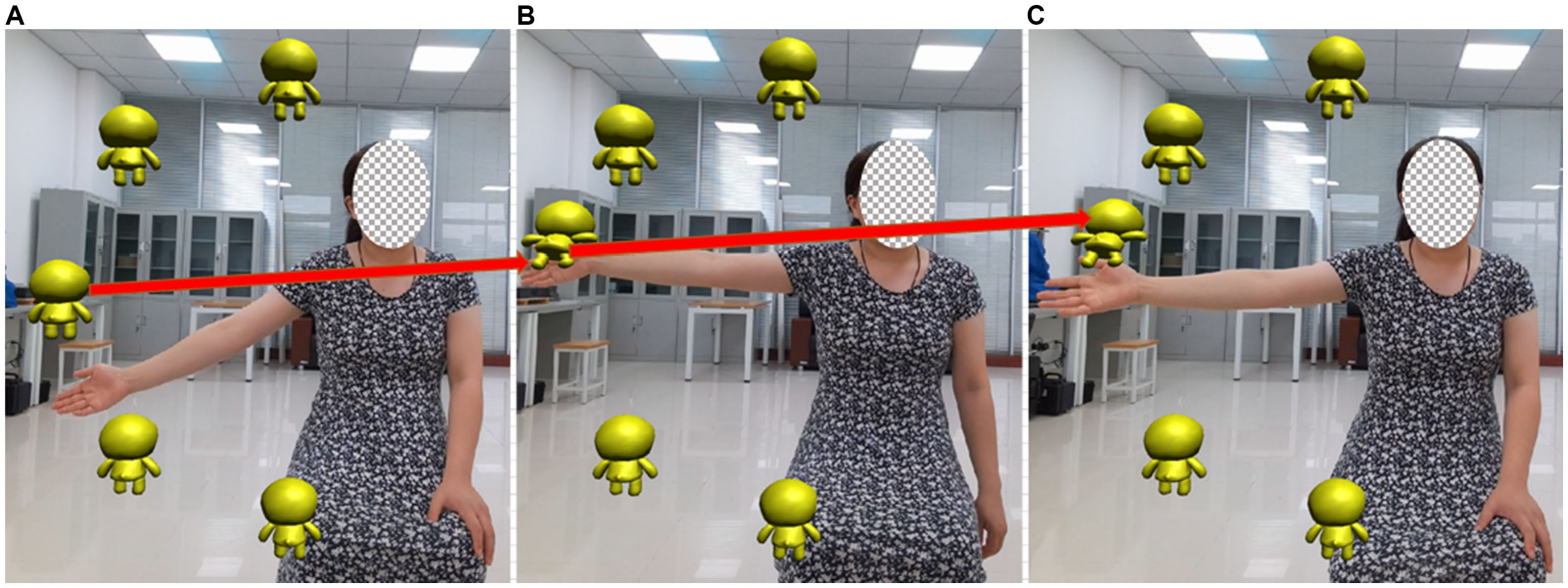
Virtual scene of motion assessment. In the rehabilitation training game, when the position of the subject’s hand coincides with the minion, the animation of the minion jumping with the sound effect is played. **(A)** The hand has not move to the minion position **(B)** Hand and minion position coincide **(C)** After the position of the hand and the minion overlap, the minion jumps and accompanies the sound effect.

Vivid virtual reality (VR) scene modeling can improve the enthusiasm and initiative of patients to participate in rehabilitation assessment. In this manuscript, a virtual scene of motion assessment was built, in which the therapist demonstrates the action, and the subjects follow the therapist to carry out the same action. The subject’s avatar was designed and the visual feedback is applied to facilitate the subjects to observe whether their movements are completed or not. Auditory feedback was used to guide related movements with a variety of sensory stimuli, to increase the feasibility of the virtual environment demonstration. The rehabilitation training game is shown in Figure 4, when the position of the subject’s hand coincides with the minion, the animation of the minion jumping with the sound effect is played. The patient’s participation increased, and the patient’s tension and anxiety were relieved.

## 3. Experimental validation

**Figure 5:**
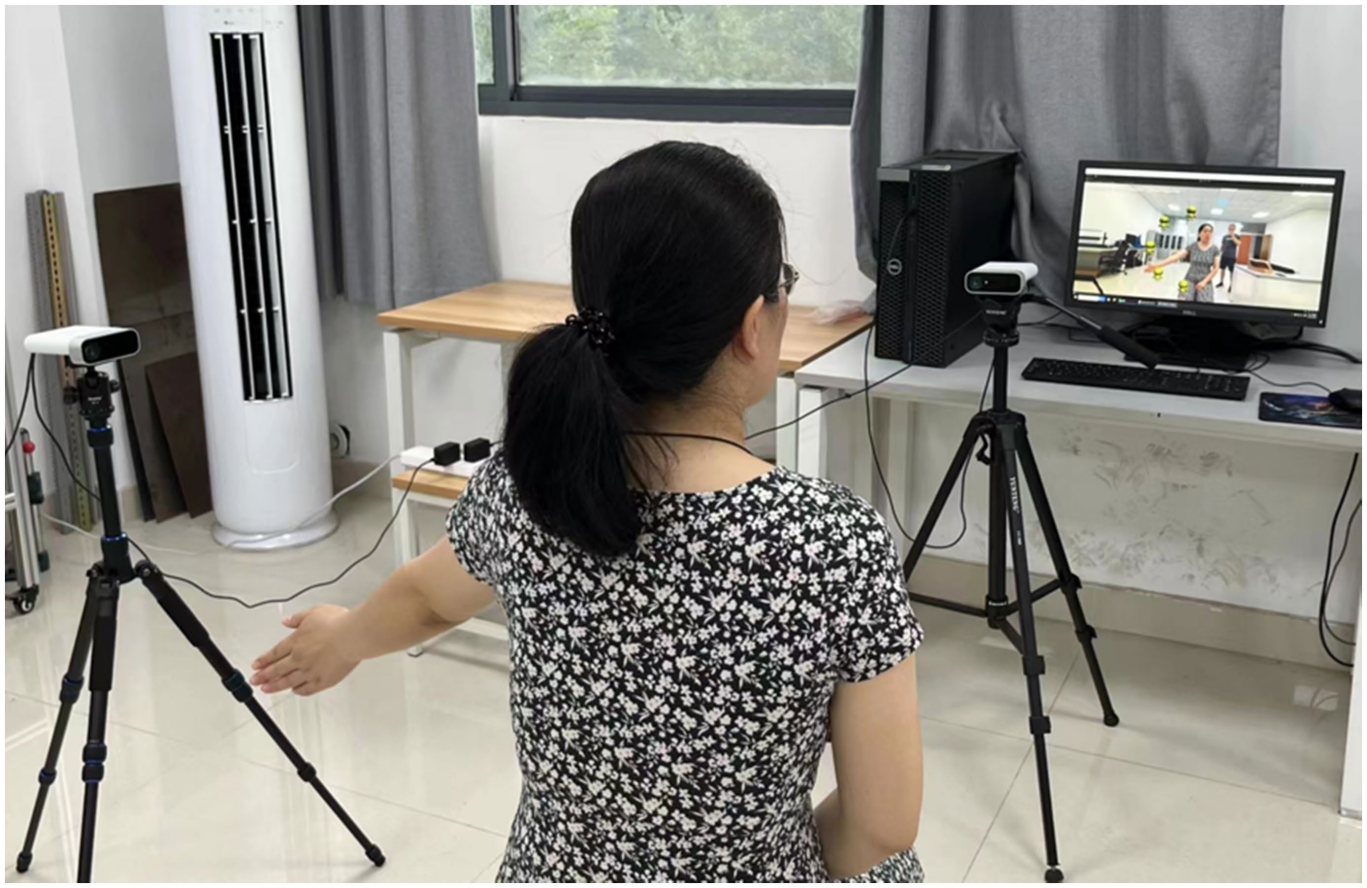
The experimental setup.

The experimental setup is as follows, two Azure Kinect depth cameras were placed on the tripod with a spacing of 2 m and an angle of 90°, and adjusted horizontally using a spirit level, as shown in Figure 5. During the experiment, the subjects were asked to perform the designed movements in front of the camera and could not rotate their bodies. To reduce the impact of accidental factors, explanations and related training were provided to the subjects before the experiment. The participants simulated the coach’s actions by watching pre-recorded videos on the display screen, enabling them to proficiently apply the assessment method before conducting relevant experiments.

This experiment recruited a total of 20 subjects, including 10 healthy individuals and 10 stroke patients. Among them, two rehabilitation physicians from the rehabilitation department of Nanjing Tongren Hospital voluntarily participated in the experiment. The exclusion criteria for participants in the experiment are cognitive impairment or inability to cooperate in the experiment. This work is approved by the local science and ethics committee.

## 4. Results

The data collected by Azure Kinect needs to be preprocessed to reduce individual differences, and eliminate migration and expansion during the experimental process. Median filtering can effectively eliminate isolated noise points. First, median filtering is performed on the data, and then the 6th-order low-pass Butterworth filter with a cut-off frequency of 30 Hz is used to filter again.

### 4.1. Model optimization result

**Figure 6:**
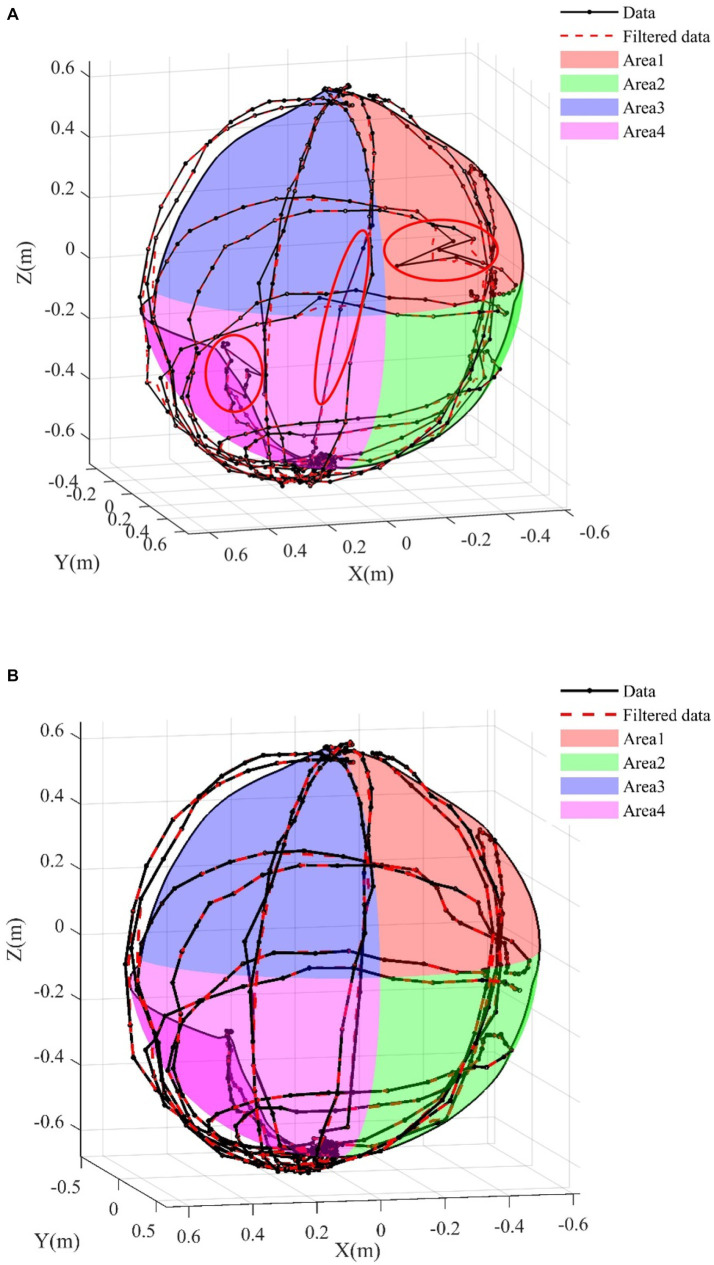
Reachable workspace results. **(A)** Raw data. **(B)** Data after rigid model.

According to the reachable workspace and proximal measurement in the exercise protocols, the validity of the optimized rigid body model is verified through the data of 10 healthy people. Taking the right upper limb as an example, the reachable workspace and its area of the upper limb was calculated by reference (Bai and Song, 2019). The brief description is as follows: the center of the upper limb workspace is at the shoulder joint, the motion trajectory is fitted using the least squares method, coordinate transformation is performed, Alpha Shape algorithm is used to locate the maximum boundary of the trajectory, Catmull-Rom spline interpolation is used to smooth the boundary, coordinate transformation is performed again, surface blocks are selected, the surface area is calculated, and normalization is performed. The reachable workspace is divided into four quadrants, with the first quadrant (blue) located on the inner side above the shoulder, quadrant 2 (pink) located on the inner side below the shoulder, quadrant 3 (red) located on the outer side above the shoulder, and quadrant 4 (green) located on the outer side below the shoulder. Figure 6 shows the reachable workspace results, A is the result measured by Azure Kinect, and B is the result optimized using the method we proposed.

In the Figure 6A the solid lines represent the original trajectory information, the dashed lines represent the preprocessed results. The red ellipse marks show some points away from the track, or even skipping points. These points are not consistent with the biological characteristics of human movement. These spots can be caused by the arms facing the camera, moving too fast, or being blocked by the torso when extended backwards.

Due to the fan-shaped measurement range of the camera, occlusion can easily occur when the arm moves between the body and the camera, or the arm extends to the back of the body. At this point, a single camera cannot detect the position information of the occluded joint. Error signals will be detected at these occlusion positions, as shown in the ellipse inside Figure 6. Occlusion positions are points with obvious jumps and abrupt changes, which can easily lead to the phenomenon of unclosed fitting boundaries in the reachable workspace.

In this study, a part of the occlusion problems can be solved by using two cameras. The other part of the occlusion problem can be optimized by adding a rigid body model. After model optimization, the number of singularities was significantly reduced, the occurrence of non-biological motion was reduced and the accuracy and stability of hand joint motion measurement was improved.

**Figure 7:**
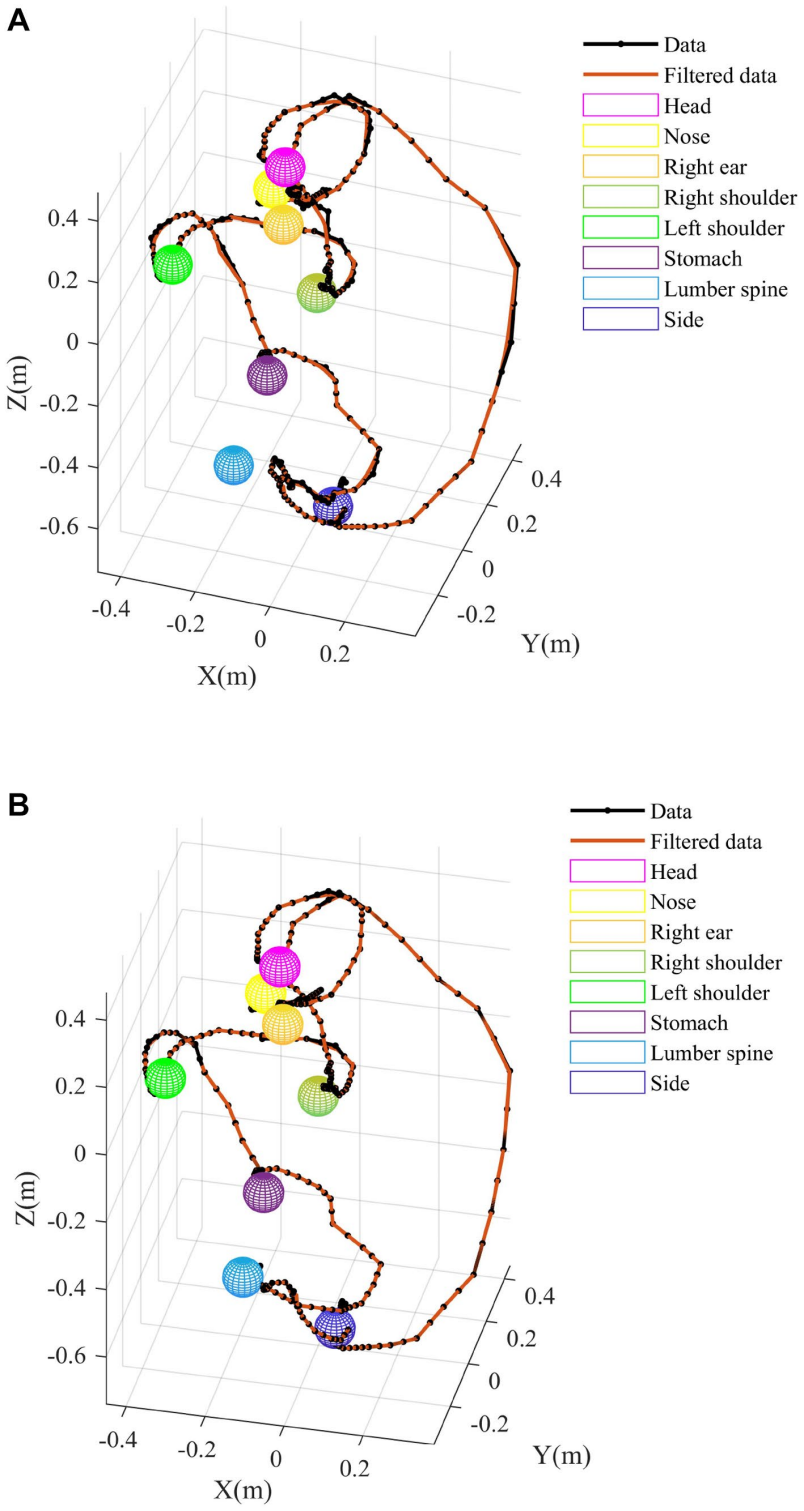
Proximal function results. **(A)** Raw data. **(B)** Data after rigid model.

Figure 7 shows the proximal function results, A is the result measured by Azure Kinect, and B is the result optimized using the method we proposed. From the comparison of Figures 7A,B, without the addition of a rigid body model, during the process of the upper limb touching the ear and the hand touching the lumbar spine, the trajectory did not reach the position of the ear/lumbar spine. After adding the rigid body model, the measurement results were improved, and the hand motion trajectory could reach the corresponding position.

Table 2 shows the Signal Noise Ratio (SNR) of the motion trajectory of raw Kinect, two Azure Kinect, and two Azure Kinect with the rigid model. The raw Kinect trajectory exhibit low SNRs, especially in the Y and Z directions of the chest joint and the Z direction of the ipsilateral shoulder joint, the signal-to-noise ratio is below 10. The SNR of the final motion trajectory measured by two Kinects has increased, but there is still an SNR of less than 10. This study applied two Azure Kinect combined with a rigid body model, the measurement results show that all directions of each joint are greater than 10, and the SNRs are greater than 20 in the elbow joint, wrist joint, and hand joint. This table indicates that the method used in this study can improve the SNR of collected signals from each joint and increase the accuracy of upper limb posture recognition.

**Table 2:** Signal to Noise ratio.

Segment	Axis	RW			PF		
		TKM	TK	RK	TKM	TK	RK
Spine chest	X	18	18	18	18	20	20
	Y	20	9	7	20	12	11
	Z	12	9	5	15	11	6
Ipsilateral neck	X	19	21	21	22	24	23
	Y	16	16	14	19	19	16
	Z	12	12	10	14	15	13
Ipsilateral clavicle	X	20	18	19	22	20	20
	Y	18	18	16	15	16	15
	Z	15	12	9	17	14	14
Ipsilateral Shoulder	X	27	20	18	20	20	18
	Y	18	11	11	19	14	15
	Z	14	9	7	16	10	7
Ipsilateral Elbow	X	26	14	14	20	14	14
	Y	25	12	11	25	15	14
	Z	28	14	14	21	13	15
Ipsilateral Wrist	X	28	17	16	29	16	15
	Y	29	14	14	27	12	11
	Z	27	17	16	28	18	17
Ipsilateral Hand	X	26	16	16	27	16	15
	Y	28	14	12	24	13	10
	Z	26	17	15	25	15	11

Two Azure Kinect optimization model (TKM), Two Azure Kinect (TK), Kinect (RK).

### 4.2. Assessment result

In rehabilitation assessment experiments, each subject underwent 5 exercises, with 30 groups tested each time and 10 groups resting for 10 min. A total of 3,000 sets of data were collected. Each action data in the dataset consists of a series of skeletal action frames. Each frame contains up to two skeletons, each with 11 skeletal nodes of the upper limbs. The data includes distal and proximal actions, with a total of 16 action categories, and each bone node has corresponding three-dimensional spatial coordinate data.

Three STGCN blocks are used. The optimization strategy of the model is the Adam optimizer, with a learning rate of 0.1, a batch size of 4, and an output space dimension of 80,40,40,80 for the LSTM layer. The model shares four LSTM layers with a dropout of 0.25.

The accuracy of the assessment model is measured using Mean Absolute Deviation (MAD), Root Mean Square Error (RMSE), and Mean Absolute Percentage Error (MAPE). The lower the error, the higher the accuracy of the model. The model was trained and predicted 10 times, and the obtained MAD, RMSE, and MAPE are recorded simultaneously. Finally, the average of the 10 results is taken to ensure the reliability of the results.

Each joint plays a different role in limb movement. Attention-guided graph convolution is used to extract spatial information, and each joint is processed differently based on the spatiotemporal frames, increasing the impact of different joints on the evaluation results. STGCN based on an attention mechanism makes the evaluation results more accurate, and can also provide guidance for rehabilitation and strengthen the training of important joints.

**Figure 8:**
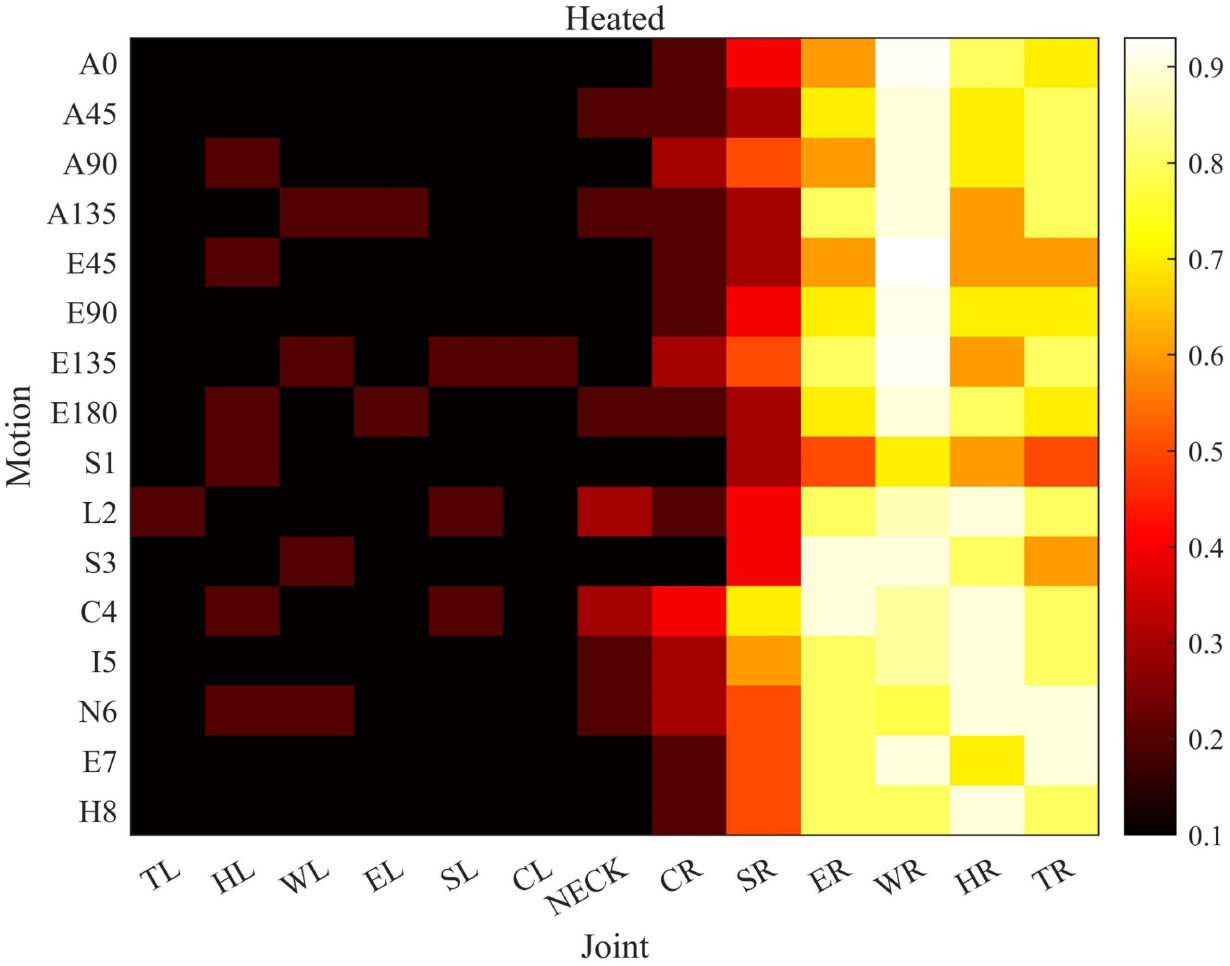
The role of joints in different movements. (The importance increases sequentially from 0 to 1. The vertical axis represents 16 evaluation actions, A * and E * represent the reachable workspace actions respectively, S1-H8 represents the proximal actions. The horizontal axis is an abbreviation for the names of each joint, from left hand to neck and then to the fingertip of the right hand).

Figure 8 shows the impact of each joint on different assessed movements. As can be seen from the figure, in the movement of touching the nose with the right upper limb, the left joints of the body participate less in the movement and show lower importance. The degree of participation of the right joints varies, such as higher participation of the elbow joint and hand joint, and lower participation of the sternoclavicular joint. The wrist joint plays a crucial role in measuring the entire reachable workspace. The importance of different joints in different movements varies.

Table 3 compares the performance of our proposed model, a single Azure Kinect and two Azure Kinect algorithms combined with STGCN. MAD, RMS, and MAPE are analyzed. It is obvious from the table that our proposed model finally gets the lowest evaluation error.

**Table 3:** Assessment results.

Metric	Our Methods	STGCN with two Azure Kinect	STGCN with one Azure Kinect
MAD	1.021	2.757	5.378
RMSE	1.180	3.259	8.671
MAPE	3.973	5.875	25.092

## 5. Discussion

(1) Improve posture recognition accuracy

Accurate recognition of posture is key to the rehabilitation assessment of upper limb motor function using posture. However, human posture recognition is very complex and the accuracy is difficult to be guaranteed. Occlusion is easy to occur when using a single Kinect (Han et al., 2016) only used a one-generation Kinect to collect the reachable workspace of the upper limb in Duchenne muscular dystrophy, without proposing a method to solve the occlusion problem, resulting in low accuracy in human pose recognition (Matthew et al., 2020) also used only one-generation Kinect, with an improvement of adding a model. The model had fewer degrees of freedom and does not include the degrees of freedom of the wrist and hand. The accuracy of human body recognition measurement was not high, and there was a significant error in proximal upper limb movement.

Therefore, this study proposes to use two Azure Kinects and increase the constraint of the rigid body model at the same time, in order to reduce the inconsistency of human bone connection in biology, and then improve the accuracy of posture recognition. In the experimental results, Figure.6 contains the action of touching the lumbar vertebrae by hand. It can be clearly seen that the occlusion phenomenon is very obvious in the absence of a rigid body model, especially when the upper limb moves to coincide with other joints, the trajectory of the occluded part is messy and irregular, and the joint motion trajectory does not comply with human biology. The results of different test methods in the figure can obviously show the effectiveness of the proposed posture recognition method. Due to the inability of the camera to test the rotational motion of the arm, a more abundant human rigid body model is proposed to measure human posture from both attitude and position, achieving accurate posture recognition.

(2) Improve the accuracy of the assessment model

The effective and accurate assessment of upper limb motor function can provide the scientific basis for rehabilitation training, but the existing rehabilitation assessment methods lack universality, robustness, and practical relevance. Convolutional neural networks can be used to design scientifically reasonable quantitative assessment methods, but the accuracy of the assessment results still needs further verification. On the basis of improving the accuracy of human body recognition, this study conducts rehabilitation assessment tasks to increase the accuracy of assessment model recognition. Due to the varying degrees of participation of each joint in different movements, the importance of each joint is increased based on attention mechanisms. When the right hand is active, the participation of the left joint is lower, while when the left hand is active, the participation of the right joint is lower. At the same time, the importance of each joint is calculated for both the expert therapist and the patient. The difference in joint function between the patient and the therapist is significant. By comparing the difference in joint function with the average expert therapist, it can be determined which joints can be trained more effectively to improve the patient’s rehabilitation assessment score based on the size of the difference. The difference in joint function can provide a reasonable direction for rehabilitation training for patients. Adding joint participation to a rehabilitation assessment model can achieve continuous assessment and improve the accuracy of rehabilitation evaluation.

## 6. Conclusion

This study addresses the issue of non-quantification in rehabilitation assessment, and proposes an improved STGCN based on precise upper limb posture recognition to achieve continuous quantitative rehabilitation assessment. Two Azure Kinects were used to expand the field of view, a multi-degree of freedom upper limb motion rigid body model was proposed, making the upper limb posture measurement in line with normal human biological movement. The accuracy of upper limb posture recognition is increased, and the signal-to-noise ratio of measurement is improved. By identifying the participation degree of each joint in different movements based on the self-attention mechanism, the STGCN algorithm was improved to achieve continuous quantitative rehabilitation assessment. The experimental comparison results show that the upper limb posture recognition algorithm proposed in this study can effectively reduce incorrect joint coordinates, and the rehabilitation assessment model based on improved STGCN can effectively reduce the assessed MAD and RMS and MAPE. This study provides a new approach for the quantitative rehabilitation assessment of stroke patients. In the future work, we will continue to optimize the rigid body model and improve the rehabilitation assessment method.

## Data availability statement

The raw data supporting the conclusions of this article will be made available by the authors, without undue reservation.

## Ethics statement

The studies involving human participants were reviewed and approved by Nanjing Tongren Hospital science and ethics committee. The patients/participants provided their written informed consent to participate in this study.

## Author contributions

JB was responsible for the study scheme design, data analysis, and manuscript writing. ZW was responsible for the data acquisition. XL was responsible for the experiment. XW was responsible for the revision of the manuscript. All authors contributed to the article and approved the submitted version.

## Funding

This work was supported by the Natural Science Foundation of Jiangsu Province (Grant no. BK20210930), Natural Science Research of Jiangsu higher education institutions of China (Grant no. 21KJB510039), and the Scientific Research Foundation of Nanjing Institute of Technology (Grant no. YKJ2019113).

## Conflict of interest

The authors declare that the research was conducted in the absence of any commercial or financial relationships that could be construed as a potential conflict of interest.

## Publisher’s note

All claims expressed in this article are solely those of the authors and do not necessarily represent those of their affiliated organizations, or those of the publisher, the editors and the reviewers. Any product that may be evaluated in this article, or claim that may be made by its manufacturer, is not guaranteed or endorsed by the publisher.
